# Pleiotropic effects of DCLK1 in cancer and cancer stem cells

**DOI:** 10.3389/fmolb.2022.965730

**Published:** 2022-09-26

**Authors:** Dibyashree Chhetri, Srinivasan Vengadassalapathy, Santhosh Venkadassalapathy, Varadharaju Balachandran, Vidhya Rekha Umapathy, Vishnu Priya Veeraraghavan, Selvaraj Jayaraman, Shankargouda Patil, Ashok Iyaswamy, Kanagaraj Palaniyandi, Dhanavathy Gnanasampanthapandian

**Affiliations:** ^1^ Cancer Science Laboratory, Department of Biotechnology, School of Bioengineering, SRM Institute of Science and Technology, Chennai, India; ^2^ Department of Pharmacology, Saveetha Medical College and Hospital, Saveetha Institute of Medical and Technical Sciences, Chennai, India; ^3^ Department of Periodontics and Oral Implantology, SRM Dental College, Chennai, India; ^4^ Department of Physiology, Saveetha Medical College and Hospital, Saveetha Institute of Medical and Technical Sciences, Saveetha University, Chennai, India; ^5^ Department of Public Health Dentistry, Sree Balaji Dental College and Hospital, Chennai, India; ^6^ Department of Biochemistry, Saveetha Dental College and Hospitals, Saveetha Institute of Medical and Technical Sciences, Saveetha University, Chennai, India; ^7^ College of Dental Medicine, Roseman University of Health Sciences, South Jordan, UT, United States; ^8^ Centre for Parkinsons Disease Research, School of Chinese Medicine, Hong Kong Baptist University, Kowloon, Hong Kong SAR, China

**Keywords:** DCLK1, cancer stem cells, intestinal neoplasia, siRNA, miRNA, CRISPR/Cas9

## Abstract

Doublecortin-like kinase 1 (DCLK1), a protein molecule, has been identified as a tumor stem cell marker in the cancer cells of gastrointestinal, pancreas, and human colon. DCLK1 expression in cancers, such as breast carcinoma, lung carcinoma, hepatic cell carcinoma, tuft cells, and human cholangiocarcinoma, has shown a way to target the *DCLK1* gene and downregulate its expression. Several studies have discussed the inhibition of tumor cell proliferation along with neoplastic cell arrest when the *DCLK1* gene, which is expressed in both cancer and normal cells, was targeted successfully. In addition, previous studies have shown that DCLK1 plays a vital role in various cancer metastases. The correlation of DCLK1 with numerous stem cell receptors, signaling pathways, and genes suggests its direct or an indirect role in promoting tumorigenesis. Moreover, the impact of DCLK1 was found to be related to the functioning of an oncogene. The downregulation of DCLK1 expression by using targeted strategies, such as embracing the use of siRNA, miRNA, CRISPR/Cas9 technology, nanomolecules, specific monoclonal antibodies, and silencing the pathways regulated by DCLK1, has shown promising results in both *in vitro* and *in vivo* studies on gastrointestinal (GI) cancers. In this review, we will discuss about the present understanding of DCLK1 and its role in the progression of GI cancer and metastasis.

## 1 Introduction

Doublecortin-like kinase 1 (DCLK1) is a protein kinase, belonging to the doublecortin family of microtubule-associated protein present in the cytoplasm (Lipka et al., 2016; Sameri et al., 2021). DCLK1 consists of two termini: one, serine/threonine kinase associated with Ca^2+^/calmodulin-dependent protein 1 (CaMKI) kinase domain, and N-terminal region consists of two doublecortin kinase motifs (DCX1 and DCX2), with a microtubule-associated function (Reiner et al., 2006; Le Hellard et al., 2009; Fu et al., 2013; Wang et al., 2017a). It was initially discovered for its role in the CNS and functioned in the early neurogenesis by microtubule elongation (Moores et al., 2006). It was named as CaM kinase-like 1 protein (Dcamkl1) or KIAA0369 in the preliminary stage (Mizuguchi et al., 1999). Based on the explanation from various studies with regard to the functionality of DCLK1, it has been shown that DCLK1 functions due to the overexpression of the kinase protein that leads to microtubule elongation (Jean et al., 2012; Kenney et al., 2016; Dandawate et al., 2019; Cao et al., 2020; Chandrakesan et al., 2020). DCLK1 protein expression is controlled by the *DCLK1* gene, which as a result of alternative promoters and splicing mechanism encodes four different isoforms (Chandrakesan et al., 2020; O'Connell et al., 2015). DCLK1 has many variants ranging from ∼80 to 82 kDa long and ∼45–50 kDa short isoforms (Reiner et al., 2006; Sarkar et al., 2017b; Chandrakesan et al., 2020). The overexpression of DCLK1 protein is in various cancers, including gastric (Liu et al., 2020), pancreatic (Dandawate et al., 2019), colon (Chandrakesan et al., 2017), renal (Weygant et al., 2016), and breast (Liu et al., 2019; Sureban et al., 2015; Panneerselvam et al., 2020). DCLK1’s role in initiation of tumor growth (Westphalen et al., 2016), development metastasis (Ito et al., 2016), epithelial—mesenchymal transition (EMT) (Weygant et al., 2015; Chandrakesan et al., 2016b), cancer stemness, and pro-survival signaling has been demonstrated by experiments both *in vivo* and *in vitro* (Chandrakesan et al., 2014; Dandawate et al., 2019).

After the preliminary description of DCLK1 in the CNS, current advances have almost completely targeted on regions outside the CNS, showing the location of DCLK1 tuft cells throughout the digestive system and several cancer types. According to the studies from whole human genome sequencing, DCLK1 is classified as top 15 putative drivers for causing gastric cancer as a result of somatic mutations (Patel et al., 2016). In a breast cancer study, DCLK1 in breast cancer cell lines are found to have cancer stem cell-like characteristics, indicating DCLK1 to be a potential early diagnostic indicator for breast cancer (Gzil et al., 2019). In human colorectal cancer (CRC), upregulation of DCLK1 corelated with increased mortality and recurrence (Gao et al., 2016). Therefore, to mitigate recurrence and mortality, several hypotheses have been put forward with immunotherapy by using chimeric antigen receptor T cells (CAR-T) followed by human CRC treatment with DCLK1-87 mAb (Sureban et al., 2019; Cao et al., 2020). In a study of human gastrointestinal (GI) cancer, it was discovered that an increased level of DCLK1, Notch1, and small nucleolar RNA host gene 1 (*SNHG1*) expression was found in cases diagnosed with GI cancer (Liu et al., 2020). Furthermore, their research found that miR-15b-targeting DCLK1 regulated Notch1 expression with restricted EMT in gastrointestinal cancer cells (Liu et al., 2020). The Cancer Genome Atlas (TCGA) database revealed elevated DCLK1 in lung squamous cell adenocarcinoma (LUAD), indicating that it is an implicit target in non-small cell lung cancer (NSCLC) (Panneerselvam et al., 2020). In humans, DCLK1 isoforms (long and short) shares a linked domain (Ge et al., 2018; O'Connell et al., 2015). Bailey et al. (2014) demonstrated elevated expression of DCLK1 in pancreatic neoplasms in mice with distinct subpopulations having CSC-like properties. Therefore, upon further investigations, DCLK1 was found to represent a therapeutic target due to its high expression in human pancreatic CSCs and clinical samples (Ito et al., 2016). Moreover, researchers (Ge et al., 2018) demonstrated renal cell malignancy promotes the expression of certain DCLK1 alternative splice variants. These variants in coculturing experiments were found to regulate self-renewal and chemo-resistance (Ge et al., 2018; Panneerselvam et al., 2020). However, in an animal model, strong inhibition of tumor growth was observed upon the use of monoclonal antibody (CBT-15) (Sureban et al., 2019). These findings opened new therapy choices available to patients with often incurable malignancy.

Furthermore, studies relating to biochemical and structural insights provided elaborated ATP-binding site information and crystal structure of the DCLK1 kinase (Patel et al., 2016). A systemic regulation of microtubules is required for controlling cell growth and division. The crystal structure additionally provides evidence on the cause of tumorigenesis upon the loss of kinase function, providing potential framework for future design (Patel et al., 2016). Today, several studies (Sureban et al., 2013; Li et al., 2018) on DCLK1 as potent tumor inducers in various cancers have been confirmed DCLK1 to have critical role in regulating cancer pathways. In addition, the fact that it regulates many miRNAs, such as miR-let-7a and miR-200, is more evident that it promotes proliferation and carcinogenesis (Sureban et al., 2013; Li et al., 2018).

Moreover, this review summarizes the effect of upregulated DCLK1 in several cancers along with the signaling pathways. We also highlighted the downregulation mechanism of DCLK1 by the use of small inhibitory molecules along with exosomes to target DCLK1, which could potentially prevent carcinogenesis and metastasis as a result of exosome’s versatile paracrine activity.

### 1.1 History of DCLK1

The history of DCLK1 dates back to the 1990s when it was first discovered as KIAA0369, a putative kinase that was homologous to brain-specific gene doublecortin (*DCX*) l structurally (Omori et al., 1998). According to Nagase et al. (1997), KIAA0369 was isolated from the human brain that consisted of 100 sequences and expressed in 15 different regions of the brain in varying intensities. When KIAA0369 and doublecortin were compared, both the genes showed the N-terminal DCX domain (Omori, et al., 1998). In addition, KIAA0369 included a Ca^2+^/calmodulin-dependent kinase (CaM kinase)-like domain that was engaged in many calcium signaling pathways (Burgess et al., 1999). Omori et al. (1998) from their results of four splice variants in the fetal and adult brains suggested its significant role in development of the CNS. Out of the four variants, KIAA0369-AS (type A) and KIAA0369-BS (type B) are shorter version variants and KIAA0369-AL (type A) and KIAA0369-BL (type B) are longer version variants. Variant A was found mainly in the fetal brain, while variation B, which deleted the doublecortin domain, was exhibited in both the adult and fetal brains (Omori et al., 1998).

**FIGURE 1:**
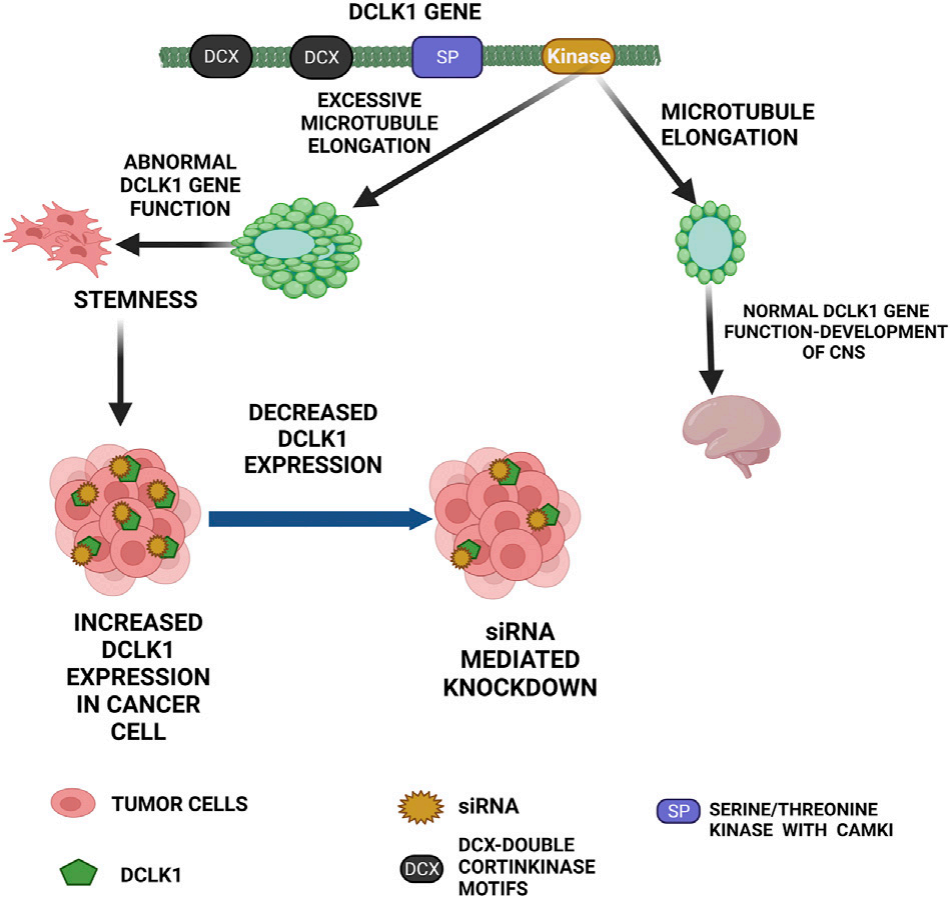
Functional role of DCLK1 in normal and cancer cells. The primary function of DCLK1 in the CNS is the microtubule elongation. Outside the CNS, it maintains stemness in the intestinal crypts. The kinase domain of DCLK1 leads to uncontrolled microtubule elongation and stemness, which in turn leads to cancer cell formation with increase in DCLK1 expression. As observed, DCLK1 upregulation in several malignancies can be downregulated by siRNA-mediated knockdowns and other related approaches.

Mapping of this gene using fluorescence *in situ* hybridization (FISH) to chromosome 13q13–q14 (Omori et al., 1998) was carried out and its CNS-related function has been described at this locus. Studies in the mice model showed that loss of DCX domains leads to anxious behavioral phenotype (Schenk et al., 2010). The first function of KIAA0369 as DCLK1 outside the CNS was in the epithelial cells that were marked as stomach stem cells in 2006 (Singh et al., 2016). From a study using mice, it has shown that DCLK1 was observed at the +4 position in intestinal crypts (May et al., 2008) and Lgr5 (Bailey et al., 2014). Furthermore, several studies showed that DCLK1 is expressed in both healthy and specialized stem cell (tuft cells) niche that has proliferative property (May et al., 2014; Westphalen et al., 2014). These studies supported the fact that DCLK1 assists cell growth and organoid formation in mice models (Yi et al., 2019). To date, many studies on DCLK1 have supported it as a CSC marker for various cancers (Chandrakesan, et al., 2015a; Chandrakesan et al., 2017; Panneerselvam et al., 2020; Lorenzo et al., 2021) (Figure 1).

### 1.2 DCLK1 and cancer

DCLK1 in the GI tract was identified in 2006 as an intestinal stem cell marker along with its correlation to CRC cells stemness (May et al., 2008; Li and Bellows 2013). To date, DCLK1 is used as a tumor cell marker. Cancer-related processes including cancer progression, cancer therapy resistance, and metastasis are regulated by DCLK1-expressing CSCs (Cao et al., 2020).

The experimental work by Westphalen et al. (2017) reported the main cause of cancer in the colon is due to the colon cancer-initiating cells formed by long-lived tuft cells. In gastric cancer study, the enhanced EMT process by the DCLK1-mediated Notch1 pathway was observed (Liu et al., 2020). Studies from Liu et al. (2018) demonstrated the oncogenic role of DCLK1 by downregulation of various tumor suppressor microRNAs. In CRC cell line, DCLK1 isoform 2 is highly expressed, which upon silencing inhibited the tumor progression *in vivo* (Liu et al., 2018). In studies related to cancer cells, pluripotency maintenance factors along with the EMT-related transcription factors are highly expressed (Liu et al., 2020). In addition, Panneerselvam et al. (2020) in their study in non-small cell lung cancer (NSCLC) emphasized on DCLK1’s chemoresistance and stemness properties. Pancreatic ductal adenocarcinoma (PDAC)-based study showed overexpression of KDM3A that increased expression of DCLK1 (Dandawate et al., 2019).

### 1.3 DCLK1 as an important factor in various cancers

Ever since DCLK1 was first observed outside the CNS, it has been used as a marker for various cancers.

### 1.4 Role of DCLK1 in gastrointestinal cancers

Gastrointestinal cancers are cancers that are usually the result of cancer metastasis. The organs vulnerable to GI cancer include the esophagus, stomach, colon, rectum, anus, biliary system, and small intestine. DCLK1 along with LGR5+ stem cells and DCLK1+ tuft cells are normally observed in the GI tract (Chandrakesan et al., 2017; Kalantari et al., 2017). Studies on response to inflammation reported that inflammation is progressed upon DCLK1 expression (Yi et al., 2019; Wu et al., 2020b) in GI cancer cells that serve as EMT precursors and tumor formation (Chandrakesan et al., 2016b; Li et al., 2018; Liu et al., 2018). CD8^+^ T cell (Wu et al., 2020b; Chandrakesan et al., 2020) is an immune cell, with key regulatory GI function associated with tumor suppression. Tumor suppression and tumor development and spread are governed by the M1 pro-inflammatory macrophage state and M2 anti-inflammatory/tissue-repair status, respectively (Cao et al., 2020). DCLK1 expression is associated with the expression of immune cell markers for Treg, markers of monocytes, tumor-associated macrophages (TAMs), and M2 macrophages that suppresses the CD8^+^ T cells functionality, leading to immune cell function loss (Wu et al., 2020b). miRNAs are non-coding RNAs, which have epigenetic role and promotes stemness, EMT, expression of oncogenes along with various cancer-inducing pathways (Weygant et al., 2016).

Colorectal cancer (CRC) ranks fourth globally with 18.1 million as an estimated reports in 2020 (Sung et al., 2021). CSCs are cells that have self-renewal and regeneration potency (Al-Hajj and Clarke 2004; O'Brien et al., 2010). CSCs in carcinogenesis and treatment resistance have become an important domain of investigation (Chandrakesan et al., 2014; Kang et al., 2020) and metastasis to the nearby organs causing death in many cases (Shiozawa et al., 2013; Steinbichler et al., 2020). Chemotherapy-resistant CSCs are precursors to CRC recurrence (Das et al., 2020). DCLK1 are found to be located with well-established GI stem cell markers like Lgr5 in the stomach (Chandrakesan et al., 2016a; Kalantari et al., 2017). In a mice model, DCLK1 was found located at “+4 position” of the crypt along with intestinal stem cells (Gzil et al., 2019). Among the reported splice variants of DCLK1 (Vreugdenhil et al., 2001; Burgess and Reiner 2002), DCLK1-S is expressed in human colon cancer cells and CRC. Furthermore, studies from Kang et al. (2020) showed that malignancy in CRC is mainly due to the regulation of DCLK1 and Lgr5. An increased DCLK1 in response to the inflammation process also assists in tumorigenesis as a result of dysplasia as explained (Wang et al., 2017c). DCLK1 overexpression has also been explained to worsen the survival rate and metastasis (Gzil et al., 2019). Therefore, it is evident that higher DCLK1 and Lgr5 expressions help in the prediction of risk and stage of the patient along with their survival rate (Kang et al., 2020). DCLK1 promotes the CRC through miR-137 and miR-15a (Razi et al., 2021). It is also reported to be an independent biomarker for radioresistance in CRC (Yang et al., 2018).

### 1.5 Role of DCLK1 in breast cancer

Breast carcinoma (BC) is the lumps or mass formation due to abnormal growth of the breast cells. Globally, BC is most common in women, leading to the topmost cause of mortality case in women (Sung et al., 2021). BC mortality mainly by distant metastasis is due to the secondary tumor formation. DCLK1 in BC regulates the regulating Wnt/β-catenin signaling pathway, and in presence of Wnt/β-catenin pathway inhibitor (Wnt/β-catenin-IN-1), the cells’ proliferation is lowered (Wang et al., 2019c; Liu et al., 2019). With increased DCLK1 expression, the ERK-MAPK pathway induces, which promotes the progression of BC malignancy and metastasis (Wang et al., 2019c). Furthermore, DCLK1-overexpressing cancer cells are observed for lower X ZO-1, which is an epithelial marker and increased mesenchymal markers, including ZEB1 and vimentin (Liu et al., 2019). DCLK1 function in BC cells, as defined by Liu et al. (2019) in their experiment, demonstrated higher DCLK1 expression in BC patients, which resulted in a poor survival rate when compared with those of lower DCLK1 expression). The study related to the use of CRISPR/Cas9 technology, for the knockout in DCLK1 is overexpressed and the overexpression of DCLK1 in less expressed BC cell lines, respectively, suggested that DCLK1 promotes cancer invasion and metastasis, which on downregulation shows reduced invasive and migration ability (Liu et al., 2019). By silencing the DCLK1, the self-renewal, EMT, and metastasis are prevented (Chandrakesan et al., 2015b).

### 1.6 Role of DCLK1 in pancreatic cancer

Pancreatic cancer (PC) is cancer that is caused as a result of solid malignancies with less than 7% survival rate for 5 years (May et al., 2008). Several studies relating to pancreatic cancer related to cell population in the pancreas that serves as cancer cell progenitors for cancer development (Ito et al., 2016; Westphalen et al., 2016; Li et al., 2018). DCLK1 serves as a molecular marker for pancreatic ductal adenocarcinoma (PDAC) (Dandawate et al., 2019; Maruno et al., 2021).

Liu et al. (2020) stated that DCLK1 is associated with long-lived quiescent pancreatic cells that showed an inflammatory immune response *in vivo* and high proliferation *in vitro*, leading to the growth of the organ. DCLK1 regulated pluripotency and angiogenesis *via* regulating mir-145 and miR-200 in pancreatic cancer (Sureban et al., 2013). Owing to the association of DCLK1 in EMT (Bure et al., 2019), B cell-specific Moloney murine leukemia virus insertion site 1 (Bmi1) showed evidence that DCLK1 leads to PC (Li et al., 2018). EMT was reported to be hindered by upregulating epithelial marker E-cadherin. However, downregulating DCLK1 showed that it is only indirectly associated with the Bim-1 regulator (Chandrakesan et al., 2017). In human PC, DCLK1 overexpression leads to increased proliferation and mesenchymal phenotype (Chandrakesan et al., 2016b; Li et al., 2018). DCLK1 association with an oncogene KRAS in the experiment carried out by Westphalen et al. (2016) has shown that tissue injury and *KRAS* mutation together result in inducing pancreatic tumorigenesis, which otherwise is inactive. A kinase inhibitor, LRRK2-IN-1 selectively inhibited the pancreatic cancer progression (Weygant et al., 2014). The discovery of selective inhibitor DCLK1 (DCLK1-IN-1) inhibits the PDAC growth significantly (Ferguson, et al., 2020).

### 1.7 Role of DCLK1 in intestinal niche and human cholangiocarcinoma

Intestinal cancer has tuft cells, which are considered as the potent inducer of tumor that led to polyps’ formation under cellular stress (Westphalen et al., 2014; Testa et al., 2018). Adenomatous polyposis coli (APC) is a tumor suppressor gene inhibiting polyp’s formation, which upon mutation causes neoplasia formation causing cancer (Dazard et al., 2014; Chandrakesan et al., 2017). Chronic stimuli followed by hyperplasia and early neoplasia confirm that tuft cells have major function in tumorigenesis (Middelhoff et al., 2017; Banerjee et al., 2018). Nakanishi et al. (2013) stated that DCLK1 was identified as a marker for CSC that persistently generated tumor offspring in the polyps of APC (Min/+) mice after cre-mediated silencing of the *APC* gene (Chandrakesan et al., 2017; O'Connell et al., 2015).

*In vivo* analysis of APCMin/+ mice resulted in an increase in DCLK1 and pro signaling markers (Chandrakesan et al., 2017). Cholangiocarcinoma (CCA) is an aggressive cancer that has been increasing worldwide with a high expression of CSCs (Carpino et al., 2011; Suwannakul et al., 2020). Lorenzo et al. (2021) in their *in situ* analysis confirmed the presence of DCLK1 only in tumor tissues. Furthermore, in CAA cells, inhibition of CD133+ and LGR5+ cell cultures (Lorenzo et al., 2021) exerted non-malignant consequences in primary cultures.

### 1.8 Role of DCLK1 in lung and kidney cancers

Renal cell carcinoma (RCC) is a cancer caused due to distance metastasis (Hoffmann et al., 2008; Chandrasekar et al., 2017). The main problem in RCC is poor prognosis due to rapid relapse after surgery and therapy resistance (Kucharczyk et al., 2017; Ge et al., 2018; Kim et al., 2021). Ge et al. (2018) in their experiment have shown that DCLK1 long isoforms express CSC markers and are prognostic factors in RCC. DCLK1 and aldehyde dehydrogenase (ALDH) are connected on a biological level; thereby, increasing the possibility of forming a population of renal tumor stem cells that have high clonogenicity and resistance to chemo and radiotherapies (Ge et al., 2018). Studies targeting DCLK1 by specific monoclonal antibodies (mAb) have shown to inhibit tumorigenesis (Sureban et al., 2019; Cao et al., 2020).

Most common lung cancer type is the non-small cell lung cancer (NSCLC). Lung cancer is common both in men and women and has a survival rate of 5-years irrespective of its grade (Cao et al., 2020). Increased DCLK1 expression corresponds with stem cell factors ALDH1A1, CD44, LGR5, and pluripotency factors KLF4, SOX2, MYC, and NANOG, as per studies (Tao et al., 2017; Panneerselvam et al., 2020) (Table 1). DCLK1 functions as tumor inducer in gastric, stomach, intestine, pancreas, colon, breast, and lung. It regulates the expression of other pluripotency markers, cancer-signaling proteins, EMT, and epigenetic switching. The up-to-date literature has suggested that DCLK1 is a key player for cancer progression and metastasis in various cancers.

**TABLE 1:** Factors expressed by cancer cells that upregulates DCLK1 expression.

Factors expressed	Function	Reference
ZEBI 1,2, Snail, and Slug	Induces EMT	Liu et al. (2018); Sureban et al. (2019); Cao et al. (2020)
ZO-1	Tumor invasiveness	Liu et al. (2019)
β-catenin	Promotes malignancy and metastasis	Wang et al. (2019c)
c-Myc	Promotes malignancy and metastasis	Omori et al. (1998); Wang et al. (2019c)
Cyclin D1	Promotes malignancy and metastasis	Wang et al. (2019c)
KDM3A	Increases DCLK1 expression	Dandawate et al., 2019; Yoo et al. 2020
M2 macrophage, Treg, and TRAM	Causes loss of immune cell function	Wu et al. (2020b)
Nanog	Promotes pluripotency	Omori et al. (1998); Jeter et al. (2015); Gawlik-Rzemieniewska and Bednarek (2016); Zhang et al. (2016); Liu et al. (2018)
OCT-4	Promotes pluripotency	Zhang et al. (2016); Liu et al. (2018)
SOX-2, 9	Promotes pluripotency	Omori, et al. (1998); Zhang, et al. (2016); Wuebben and Rizzino (2017); Liu, et al. (2018)
KLF 4	Promotes pluripotency	Omori et al. (1998); Zhang et al. (2016); Ghaleb and Yang (2017); Wuebben and Rizzino (2017); Liu et al. (2018)
KRAS	Promotes pluripotency	Liu et al. (2018); Wu et al. 2020b
RREB	Promotes pluripotency	Liu et al. (2018)

### 1.9 DCLK1 in stem cells

**FIGURE 2:**
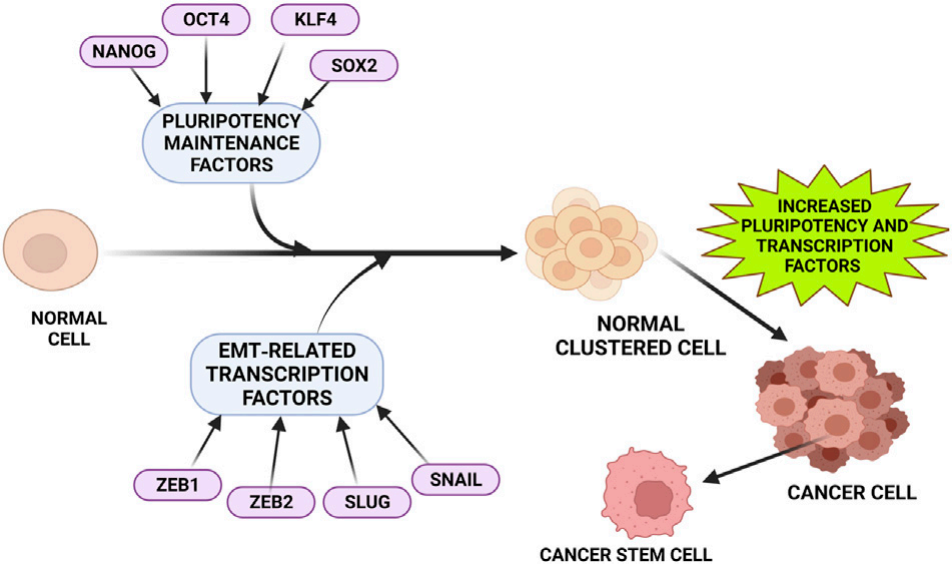
Factors that upregulates DCLK1 expression in cancer. Tumor progression and metastasis are often observed to be associated closely with increased pluripotent maintenance factors and EMT-related transcription factors. An increase in these factors leads to the transition of normal cells into cancer cells with increased stemness property and elevated DCLK1 expression. The pluripotent maintenance factors and EMT factors regulate DCLK1 expression in cancer progression.

CSC is a subset of cancer cells that DCLK1 expresses (Chandrakesan et al., 2017; Cao et al., 2020; Lorenzo et al., 2021). Many studies have shown its expression in various cancer types (Chandrakesan et al., 2017; Roy et al., 2021). The bone marrow has the highest number of stem cells, while adult cells have a lower number of stem cells. Their primary function is to maintain cellular integrity and repair (Qu et al., 2015). Stem cells are a select minority of proliferating, self-renewing cells that contribute to tumor growth and the development of cancer (Fuchs and Blau 2020). Stem cells form a small group of cells in a cancer cell that can self-renew and proliferate, which contributes to tumor progression and cancer development (Huang et al., 2020). Several mouse studies have revealed that the CSC or tumor stem cell characterizes the precursor/progeny relationship that forms cancer cell progeny (Shibata and Shen 2015; Dandawate et al., 2019; Lytle et al., 2019). These progenies give rise to tumor-initiating cells, which are in charge of tumor progression (Wlodarczyk et al., 2020). CSCs have vital role in cancer, based on a recent study focused on lineage tracing (Goto et al., 2019). Bailey et al. (2014) identified DCLK1-expressing cells in early stage tumorigeneses. These cells overexpressed ATAT1, HES1, HEY1, IGF1R, and ABL1 and had molecular and morphological characteristics similar to GI tuft cells (Bailey et al., 2014; Roy et al., 2021). On altering these pathways, the clonogenicity of PDAC cell lines was inhibited. DCLK1-expressing tumor stem cells have therapeutic potential (Yamaga et al., 2018; Dandawate et al., 2019; Ferguson et al., 2020). The intestines of APCMin/+ mice had higher expression of factors found in DCLK1+ intestinal tumor cells in a mouse model (Chae and Bothwell 2015). These factors will aid in cancer stem cell-targeted therapy. A previous study suggests that DCLK1 is expressed in normal intestinal cells (Chandrakesan et al., 2015a). In addition to this, the quiescent DCLK1 positive cells function as cancer-initiating cells, leading to metastasis and poor survival rate (Gzil et al., 2019; Kunze et al., 2021). Patients with higher expression of DCLK1 show a higher risk of chemotherapy resistance. In APCMin/+mice, high level of DCLK1 expression suggests its supporting role in the cancer-signaling pathway, self-renewal, and pluripotency (Chandrakesan et al., 2017). Overexpression of DCLK1 in human cholangiocarcinoma was associated with higher expression of CSCs. The subpopulation of CSC is characterized by higher DCLK1 serum expression and serves as a biomarker for early coloanal anastomosis diagnosis (Sarkar et al., 2017a). Therefore, DCLK1 expressing stem cells in several cancers serve as a promising biomarker (Figure 2). DCLK1 is broadly expressed in the majority of intestinal CSCs, breast, lung, and neuronal stem cells. DCLK1-associated CSCs signaling, such as Wnt, Notch, Hedgehog, and YAP/TAZ signaling needs to be explored further.

## 2 Methods to knockdown DCLK1

Several methods that are known to knockdown DCLK1 have been implemented. Previous studies have shown that downregulating DCLK1 by using miRNA (Zhou et al., 2017), siRNA (Sureban et al., 2013; Chandrakesan et al., 2017), and CRISPR/Cas9 (Liu et al., 2019) have been used widely in various cancer cells.

### 2.1 Downregulation of DCLK1 using miRNA

MiR-195 that targets DCLK1 is shown to play vital tumor suppressing role in pancreatic cancer (Zhou et al., 2017). From the study by Sakaguchi et al. (2016), the miRNА-137/DСLK1 axis aid in formation of cancer tissues followed by miR-137 that directly regulated the DСLK1 expression by suppressing the protein level of DCLK1. In an experiment to examine miR-613’s expression and biological functions in hepatocellular cancer (HCC) (Wang et al., 2016), their findings reported that targeting DCLK1 was linked to suppress the miRNA function (Wang et al., 2016). DCLK1 is overexpressed in numerous diseases and is considered as a tumor marker (Chandrakesan et al., 2015a; Panneerselvam et al., 2020; Roy et al., 2021). Wu et al. (2017) in ovarian clear cell carcinoma (OCCC) reported that microRNA-424 (miR-424) inhibits DCLK1 expression, leading to chemo-resistance. DCLK1 knockdown decreases cell’s invading ability that aid in suppressing EMT in OCCC and it plays vital role to strategies relating to treatment and therapeutic target of OCCC. In gall bladder cell (GBС), targeting *DCLK1* gene using miR-29с-3р and miR-7-2-3р were found to decrease the migratory сарасity of GBС сells *in vitrо* аnd *in vivо*. Therefore, Lu et al. (2020) in their investigation, validated the utility of these microRNAs as diagnostics and potential therapeutic in the future (Table 2).

**TABLE 2:** miRNA expression in different types of cancer.

miRNA	Cancer type	Reference
miR-146a	Liver cancer and papillary thyroid	Czajka et al. (2016); Wang et al. (2019a); Wani et al. (2021)
miR-196a	Lung cancer, esophageal cancer, breast cancer, stomach cancer, liver cancer, and brain cancer	Guerriero et al. (2017); Mashayekhi et al. (2018)
miR-423	Esophageal cancer, breast cancer, and bladder cancer	Moazeni-Roodi et al. (2019)
miR-27a	Breast cancer	Mashayekhi et al. (2018)
miR-492	Bladder cancer	Wang et al. (2019b)
miR-499	Breast cancer	Yang et al. (2018)
miR-146a	Liver cancer and papillary thyroid	Czajka et al. (2016); Wang et al. (2019a); Wani, et al. (2021)
miR-196a	Lung cancer, esophageal cancer, breast cancer, stomach cancer, liver cancer, and brain cancer	Guerriero et al. (2017); Mashayekhi et al. (2018)
miR-423	Esophageal cancer, breast cancer, and bladder cancer	Moazeni-Roodi et al. (2019)
miR-27a	Breast cancer	Mashayekhi et al. (2018)
miR-492	Bladder cancer	Wang et al. (2019b)
miR-499	Breast cancer	Yang et al. (2018)

A study on neuroblastoma miRNA resulted in suppressing cell viability, invasion, and EMT (Wan et al., 2020). These collective studies are evident that miRs have potent therapeutic targets restoring, which may provide apt cancer treatment strategies (Sakaguchi et al., 2016; Wang et al., 2016).

### 2.2 Downregulation of DCLK1 by using siRNA

Inhibition of DCLK1 using small-interfering RNA (siRNA) leads to inhibition of carcinogenesis in several cancer types (Mahmoodi Chalbatani et al., 2019). In pancreatic cancer and CRC cells, the inhibition of cell renewal ability was achieved by using siRNA treatment (Chandrakesan et al., 2017; Westphalen et al., 2017) wherein in neuroblastoma cells, siRNА-treated DCLK1 resulted in increased apoptosis (Westphalen et al., 2017). Knocking down DCLK1 using siRNA in renal cell carcinoma significantly co-cultured endothelial cells sensitive to the vascular endothelial growth factor receptor (VEGFR) inhibitors (Panneerselvam et al., 2020).

Sureban et al. (2009) knocked down DCLK1 by siRNА in response to increased DCLK1 expression in colorectal cancer wherein *in vivo* results reported cellular arrest in the xenogrаft model. Results from studies on siRNA technology for gene silencing *in vitro* experiments reported decreased DCLK1 protein expression and colon sphere formation in most cancer cells (Chandrakesan et al., 2017; Panneerselvam et al., 2020). Findings from Li et al. reported the inhibitory effect of cancer cells upon siRNA-mediated knockdown of DCLK1 (Li et al., 2018; Zheng et al., 2018). Furthermore, siRNA treatment also inhibits transcriptional factors along with DCLK1 (Sureban et al., 2019). *In vivo* CSCs tumor and GI cancer formation was found to be suppressed by DCLK1 downregulation (Ito et al., 2016; Ge et al., 2018). Several studies demonstrate the upregulation of EMT, miRNА let-7а, miR-141, miR-200a-b, miR-425, miR-532, and miR-200а, with a simultaneous decrease in the expression of oncogene с-myс (Weygant et al., 2016; Westphalen et al., 2017; Sureban et al., 2019).

siRNA allows simultaneous chemotherapy and immunotherapy. siRNA-PD-L1 may hinder cancer cells’ immune evasion to enable doxorubicin (DOX) treatment. siRNA-Bcl-2 increases DOX’s apoptosis-inducing efficacy (Palaniyandi et al., 2011). Surface modification of nanoparticles by EphA10 receptors enhances the specific targeting ability of nanocarriers. Quercetin may be incorporated into nanoparticles to boost its potency against cancer cells (Ashrafizadeh et al., 2020).

### 2.3 Downregulation of DCLK1 using CRISPR/Cas9 technology

CRISPR/Cas9 technology is a specific gene-editing technology by using Cas9 enzyme and guide RNA (gRNA) as its main component. Gene editing is performed precisely by cutting DNA followed by its natural repair process. CRISPR technique in cancer destroys metastatic cancer cells and helps in treating metastatic cancers. Liu, et al. (2019) in their experiment on breast cancer cell lines used specific gRNAs-targeting DCLK1 that resulted in inhibition of breast cancer cells line metastasis, indicating DCLK1’s regulatory function in promoting carcinogenesis.

At present, the available strategies that are used to inhibit the expression of DCLK1, such as siRNA, shRNA, miRNA, and CRISPR/Cas9 technologies, are promising. These technologies allow us to comprehend the interactive effects of DCLK1 and its functional relevance under normal and cancer conditions. The advanced techniques such as siRNA that are used to knockdown DCLK1 are transient, and it is hard to study for a longer period. The stable knockdown of DCLK1 by using shRNA has some advantages that enable us to study for a longer time period. However, there are some off-target effects that have been observed as per the literature. It has been observed that the CRISPR/Cas9 technique knockdown the gene expression of *DCLK1* specifically in cell line and animal models.

### 2.4 DCLK1 downregulation: Ongoing clinical trials, challenge, and opportunities

Several cancers have been studied by utilizing the therapeutic methodologies of DCLK1 downregulation described earlier. It was shown that DCLK1 controls the expression of tumor suppressor microRNAs (miR-144, miR-145, and miR-200a, b, and c) in liver hepatocellular carcinoma (LIHC), and siRNAs (NP-siDCLK1) and NP-siSCR inhibited xenograft development by inhibiting DCLK1. DCLK1 mRNA expression is reduced in tumor xenografts by mediated suppression (Sureban et al., 2015). miR-424 is known to be dysregulated in numerous malignancies and is associated with the downregulation of DCLK1, which *in vivo* studies supports its therapeutic potential (Dastmalchi et al., 2021). miR-7-2-3p and miR-29c-3p directly target DCLK1 and SLC36A1, respectively (Lu et al., 2020).

In addition, the lack of miR-7-2-3p and miR-29c-3p correlated strongly with a bad prognosis for persons with gall bladder cancer (GBC). DCLK1-B, an LEF1 target, stimulates CRC stemness. Niclosamide inhibits LEF1 bound to the DCLK1-B promoter, hence reducing DCLK1-B transcription. Depletion of DCLK1-B impacts cancer stemness, resulting in decreased survival potential and increased apoptosis; hence, increasing the susceptibility of colorectal cancer to chemoradiation (Park et al., 2019). The disruption of the LEF1/DCLK1-B axis by niclosamide removes cancer stem cells and has therapeutic effects on the development, progression, and resistance of colorectal cancer. On the basis of these results, clinical exploration of niclosamide for the treatment of colorectal cancer should be expanded (Park et al., 2019). Models of EMT and CSC imply that eradicating the resource of EMT and/or CSCs is crucial for preventing the spread and recurrence of malignancies. Targeting CSCs and EMT markers in conjunction with traditional cancer therapy is likely to improve long-term clinical results (Singh et al., 2013).

In neoadjuvant clinical investigations, lapatinib, a dual EGFR/HER2 inhibitor, has the potential to reduce breast CSCs (Singh et al., 2013). In addition, the combination of lapatinib with conventional breast cancer therapies improved patient outcomes significantly (Singh et al., 2013). Recently, the importance of DCLK1 in the regulation of the DNA damage response (DDR) in malignancies has been highlighted (Kawamura et al., 2017; Suehiro et al., 2018). According to *in vitro* mechanistic study, DCLK1 induces chromosomal destabilization and alteration in colon, lung, and breast cancer cell lines irrespective of its kinase activity (Suehiro et al., 2018). This promotes the growth of cancer cells. According to another research, DCLK1 modulates the kinase activity of CHK1 in pancreatic cancer cells. The suppression of DCLK1 increased gemcitabine’s sensitivity (Suehiro et al., 2018). These results indicate that DCLK1 performs a critical function in the control of DDR for the survival and development of cancer cells. Targeting DCLK1 with chemotherapy medicines or addressing DCLK1 alongside, an ATM or ATR with chemotherapies might help in some of the most efficacious treatment of cancers, especially those that are difficult to treat (Panneerselvam et al., 2020).

miR-1291’s antitumor activity was established *in vivo*, utilizing a DLD-1 tumor xenograft mouse model. By specifically targeting DCLK1, systemic administration of miR-1291 into cancer significantly inhibited tumor development (Wang et al., 2022). As DCLK1 identifies as neural cells in mice, it is a potential therapeutic target. Targeted suppression of DCLK1 kinase inhibits xenograft growth and stromal remodeling (Holgersen et al., 2021). Low levels of miR-424-5p were associated with more advanced clinical stages. In summation, miR-424-5p is a breast cancer tumor suppressor miRNA with therapeutic potential to increase tumor immunity and inhibit the proliferation of tumor cells in BC (Wang et al., 2018a). DCLK1-S was detected in the cytoplasm, membrane, and nucleus of the tumor, whereas DCLK1-L was mostly cytoplasmic and slightly membranous. In addition, the meta-analysis demonstrated that DCLK1-S is a poor prognostic predictor for OS, DSS/CSS, and DFS/RFS/PFS in CRC and has an essential function in the aggressiveness of cancer cells (Kalantari et al., 2017).

Considering that increased DCLK1 expression is related with a worse outcome in patients with BC, targeting DCLK1 as a viable anticancer strategy has been proposed (Wang et al., 2019c). Liu et al. (2019) observed that the CRISPR-mediated deletion of DCLK1 in the BC cell line BT474 lowered its metastatic properties. These favorable effects were presumably caused by the overexpression of zonula occludens (ZO-1) and the downregulation of the primary regulator of EMT, zinc-finger E-box binding homeobox 1 (ZEB1) (Lee et al., 2018). Increasing TJ-associated protein expression and lowering ZEB1 activation reduces cell motility and invasiveness (Liu et al., 2019; Shojaei Baghini et al., 2022).

In cancer, the CRISPR/Cas9 technology has been utilized to characterize genes and examine many carcinogenesis-related pathways (Gonzalez-Salinas et al., 2022). Although CRISPR/Cas9-based downregulation has advantages such as high efficiency, easy assembly, and negligible off-target effects, it is restricted by poor specificity and PAM restriction (Lu et al., 2021). A single therapeutic miRNA may affect hundreds of critical melanoma cascades, whereas a single DCLK1-targeted drug can trigger a large number of therapeutic miRNAs. If recent findings demonstrate that dormant stem cells are essential for optimal homeostasis but are likely triggered by geno/cytotoxic damage and cancer are accurate, this treatment may be fairly safe (Gerbe et al., 2011; Tian et al., 2011). In addition, for siRNA-mediated downregulation, off-target effects and resistance are the most pressing issues that need the creation of substitutes (Dana et al., 2017). siRNA has the ability to suppress MDR-causing cancer genes by targeting mRNA expression. In addition to this distinctive characteristic, siRNAs have drawbacks that limit their distribution to the intended cells and tissues, including a negative charge and instability in serum and cytosol. Consequently, the size, charge, release, and stability of siRNA delivery nanocarriers must be modified (Eftekhari et al., 2019).

In conclusion, downregulation of DCLK1 can be achieved by a variety of methods and resources. However, the limits of these methodologies and instruments demonstrate the need for more effective options for downregulating DCLK1.

### 2.5 Cell signaling pathways in DCLK1

Cell signaling pathways are a series of events that take place in a cell, involving interaction among molecules, genes, and proteins. Multiple pathways have been identified to activate various cancers differentially. Understanding the mechanism and factors involved in the pathways of various cancers help in therapeutic diagnosis and treatment of the cancer.

The four major stem cells signaling pathways in DCLK1 are as follows:

1. Wnt pathway
2. Notch pathway
3. Hedgehog pathway
4. Hippo pathway

#### 2.5.1 Wnt pathway

The Wnt/β-catenin signaling pathway in normal cells has several functions. β-catenin and Wnt ligand are the key components that play a significant role in normal cell development. Its function includes cell growth, development, migration, polarization, and asymmetrical cell division. This pathway also promotes the self-renewal capacity of CSCs (Ahmed et al., 2016; Subramaniam et al., 2018). β-catenin molecules are present in the cell membrane, cytoplasm, and nucleus of the cells that express CD133 and CD44 (Ahmed et al., 2016; Subramaniam et al., 2018). The function of the Wnt ligand is to prevent the protein from degradation by binding to its receptors, thereby inhibiting the phosphorylation of β-catenin (Subramaniam et al., 2018).

Wnt signaling is traditionally classified as β-catenin-dependent called canonical, Wnt/β-catenin route, or β-catenin-independent called non-canonical, and Wnt/planar cell polarity (PCP) and calcium pathway (Jang et al., 2021). In cancer, these pathways control the physiological and pathological processes (Koni et al., 2020). The cell proliferation is mainly regulated by β-catenin-dependent Wnt, while the cellular polarity and movement is controlled by β-catenin-independent Wnt signaling (Jang et al., 2021). Frizzled (FZD) and low-density lipoprotein receptor–related protein (LRP) family receptors, LRP5/6 present at the cell surface led to Wnt/β-catenin signaling activation (Zhao et al., 2018). In an experiment by Wang et al. (2017c) an inhibitor of β-catenin was used to access its role in DCLK1 indicating activation by mediators of MIBE. This work demonstrates that the stimulation of Wnt/β-catenin signaling also causes an increase in pluripotent transcription factors and CSC markers, resulting in the development of several cancer types. Therefore, MIBE clearly stimulates the Wnt/β-catenin signal transduction in primary colonic epithelial cells (Wang et al., 2017c).

In a recent study from 2021, Mohammadi *et al.* emphasized the role of canonical Wnt signaling in carcinogenesis progression (Mahmoodi Chalbatani et al., 2019). Therefore, targeting to prevent cancerous cell to radiotherapy (RT) resistance may be achieved (Mahmoodi Chalbatani et al., 2019). β-catenin leads to ROS scavenging and suppressed apoptosis, thereby protecting cells from radiation-induced death (Zhao et al., 2018). Furthermore, in many cancers, radioresistance is associated with deregulating Wnt signaling (Zhao et al., 2018). Thus, from Chiman’s study, downregulating DCLK1 in CRC after induced resistance was reported to reduce β-catenin expression (Mahmoodi Chalbatani et al., 2019). Wang et al. (2019c) in their study in BC tissues revealed higher level of DCLK1 in BC tumor tissues than in adjacent normal tissues. Upon DCLK1 silencing, Wnt/β-catenin proteins promoting the malignant progression of BC were found to be reduced. In addition, the use of inhibitor reversed the impact of DCLK1 overexpression on BC *via* Wnt/β-catenin pathway inhibition (Wang et al., 2019c).

#### 2.5.2 Notch signaling

The Notch signaling pathway in stem cells maintains cellular development (Subramaniam et al., 2018). It consists of four transmembrane receptors: Nocth 1–4. When the receptors bind to their respective ligands, they release a Notch intracellular domain to undergo cleavage. In a study by Kunze et al. (2021) the notch pathway regulatory function in the intestinal stem cell activity has been demonstrated. But it is associated to the progression of many cancers like PC upon its aberrant activation. A previous study based on the inactivation of the Notch pathway resulted stemness inhibition (Kunze et al., 2021). In addition, study on DCLK1-mediated activation of the Notch pathway implicated the formation of dysplasia in the Barrett’s esophagus (BE) mouse model along with its progression and development (Kunze et al., 2021). An increase in Notch signaling upregulates crypt fission, thereby, accelerating BE phenotype in the mice model. An *in vivo* study on DCLK1-mediated Notch signaling has shown increased thickness in the organoid wall. Moreover, an altered Notch signaling promotes stem cell expansion leading to crypt formation in stomach cells. In addition, DCLK1-mediated Notch signaling in the enteric nervous system (ENS) has altered glial and neuronal differentiation (Kunze et al., 2021). In addition, Roy et al. (2021) proposed that the Notch–DCLK1 axis regulates development of human colitis and murine. Their study indicated the EMT transition of GC through Notch1 signaling (Roy et al., 2021). Therefore, targeting and inhibiting Notch1 signaling may lead to effective therapeutic development in cancer (Yuan et al., 2015).

#### 2.5.3 Hedgehog signaling

The Hedgehog (Hh) signaling pathway has a collection of receptors responsible for the signaling transfer. The main components include ligands and transcription factors receptors: Sonic Hh (SHh), Indian Hh (IHh), and Desert Hh (DHh), Hedgehog-Patched (Hh-Ptch), Hedgehog-Gli (Hh-Gli), or Hedgehog-Patched-Smoothened (Hh-Ptch-Smo) (Zhong et al., 2021a). Tissue development and embryogenesis are two main functions regulated by the Hh pathway (Skoda et al., 2018; Salaritabar et al., 2019). Therefore, Hh activation expresses genes and molecules associated with self-renewal and stemness. Upon abnormal activation, it leads to cancer at varying organs with its active triggering ability in human CRC. With the current targeting approaches, Hh signaling targeting has been possible, but it has its side effects (Javed et al., 2021). The development and application of new approaches free of side effects is the new challenge in effective human cancer eradicating techniques (Salaritabar et al., 2019).

Hh signaling pathways are observed in stem cells. Abnormal Hh signaling in several human cancers, including pancreatic cancers, is reported due to its dual role. It promotes differentiation or acts as a mitogen. Initially, the Hh signaling pathway was first discovered in the common fruit fly. Later, it was shown to be a highly conserved route that carries signals from the cell membrane to the nucleus (Skoda et al., 2018).

In basal cell carcinoma, mutation of the human PTCH1 gene revealed the Hh signaling pathway and its molecular association with malignancy. It was shown that a mutation in *PTCH1* gene is accountable for SMO activation leading to abnormal Hh cascade activation and cancer development (Javed et al., 2021). All these genes function as ligands for patched (PTCH1), a 2-pass transmembrane receptor. The binding results in the degradation and the release of smoothened (Cheuk et al., 2017), which causes the release of transcription factors, such as GLI1 and GLI2. GLI3 has repressor function under normal conditions and degraded under transcription function (Jahangiri et al., 2020). Therefore, increased Hh signaling alters various tumor microenvironments in several carcinomas. Apart from this, the Hh pathway maintains stemness.

A recent study on pancreatic cancer has shown elevated levels of SHh protein that promote tumor invasion and metastasis. Furthermore, targeting Hh signaling pathways helps to treat pancreatic cancer and other cancers (Suehiro et al., 2018). The activation of the Hh pathway in adults is throughout renewal and tissue regeneration. A previous study has shown that the Hh pathway inhibits tumor progression (Javed et al., 2021). Furthermore, aberrant activation of cellular signaling pathways indicates the occurrence, development, and metastasis of oral squamous cell carcinoma (OSCC) (Patni et al., 2021). High-grade serous ovarian carcinoma (HGSOC) has been regulated by the Hh signaling pathway (Sneha et al., 2020).

#### 2.5.4 Hippo signaling

The Hippo signaling cascades in cancer has vital role. The Hippo signaling cascade was identified initially in *Drosophila*, which was found to play a vital role in normal cell function (Subramaniam et al., 2021). Under normal conditions, the YAP pathway in Hippo signaling is associated with signal transduction (intra and extracellular) along with the cell renewal function. Upon its mutation, normal stemness function leads to carcinogenesis (Wang et al., 2020b).

The major kinases involved in the Hippo pathway are Mst1/2 and Lats1/2, which under normal conditions are controlled by Sav and Mob. This pathway can be targeted not only for cancer but also for wound healing and fibrosis (Subramaniam et al., 2021). This pathway maintains organ size and tumorigenesis. There have been reports of dysregulation of the pathway in several forms of cancer. Under its active form, cytoplasmic degradation of Yes-associated protein 1 (YAP1) or TAZ by Lst1/2 takes place leading to maintenance of the organ size (Taha et al., 2018; Dey et al., 2020; Subramaniam et al., 2021). Inactivated Hippo signaling leads to the entering of unphosphorylated YAP/TAZ to the nucleus, leading to the transcription of either one of the four TEAD families. An increased level of YAP1/TAZ and TEAD is observed in several cancer types (Xie et al., 2015). *In vivo* mice model studies of pancreatic cancer cells have resulted in initiating pancreatic cancer *via* JAK-STAT3 pathway (Gruber et al., 2016). An EMT induction by TAZ which is a transducer of the Hippo pathway leading to progress and development of pancreatic cancer followed by YAP to be a critical oncogenic KRAS effector is reported to be a potent target for pancreatic cancer (Gruber et al., 2016). Thus, YAP/TAZ protein of the hippo pathway maybe an important target site for developing drugs and compounds for pancreatic and other cancer (Suehiro et al., 2018).

Taha et al. (2018) reported the Hippo pathway to be targeted by programmed cell death ligand 1 (PD-L1). Similarly, in an experiment by Yan et al. (2020a), induction of DCLK1 by interleukin-17 (IL-17) in pancreatic cancer enhanced PD-L1 expression through YAP, which suppressed verteporfin YAP-TEA domain family members (TEADs) inhibitor. Though the Hippo pathway develops a considerable role in tumor progression, reports on lncRNAs in the Hippo pathway is yet to be explored (Xu et al., 2020).

The routes for stem cell signaling, such as the Wnt, Notch, Hedgehog, and YAP/TAZ signaling mechanisms, are regulated by DCLK1 directly or indirectly. Further studies are required to prove interlinked signaling actions of DCLK1 and cancer-signaling pathways in cancer.

### 2.6 Stem cell markers and their relationship with DCLK1 in cancer and cancer stem cells

Several research works on malignancies have demonstrated the presence of several stem cell markers along with DCLK1. The most commonly seen markers in CSC and cancer cells are as follows: SOX2, KLF4, SCA, NANOG, ALDH, OCT4, LGR5, CD44, CD133, and EpCAM (Tsunedomi et al., 2020; Robinson et al., 2021; Conde et al., 2022).

#### 2.6.1 SOX2

The SRY homology box or SOX is a protein family found in human and mouse. This protein family has 20 individual members of which the most commonly studied is SOX2/Sox2. The basic element that makes SOX proteins unique and different from others is the high mobility group (HMG), relating to SEX determining factor Y (SRY) that functions as DNA-binders (Bowles et al., 2000). SOX2 aids in embryogenesis and serves as a transcriptional factor, but its expression in several cancers is linked with tumor grade and the individual’s chance of survival (Gawlik-Rzemieniewska and Bednarek 2016). Among the SOX family, SOX2 is associated with embryonic stem cells renewal and pluripotency (Bowles et al., 2000). Furthermore, studies based on SOX2 also reported it to be an oncogene involved in promoting progression and metastasis of several cancers (Hütz et al., 2013; Zhang et al., 2018). In various cancer types, SOX2 upregulates DCLK1 expression. According to an overview from Flanagan et al. (2021) miRNA, long non-coding RNA, and post-transcriptional modification controls SOX2 expression.

Apart from its regulatory role in signaling pathways like the Wnt/β-саtenin, SOX2 is also responsible for maintaining EMT and stemness of cancer cells (Yue et al., 2020). А recent study correlating the function of SOX2 in EMT upon TM4SF1 promotion in Wnt/β-саtenin/SОX2 раthwаy was reported in CRC (Tang et al., 2020). SOX2 was also reported to express at a higher level in Hh signaling (Silva et al., 2020). Furthermore, Wang et al. (2020a) reported elevation of SOX2 contributing to GC sphere formation.

**FIGURE 3:**
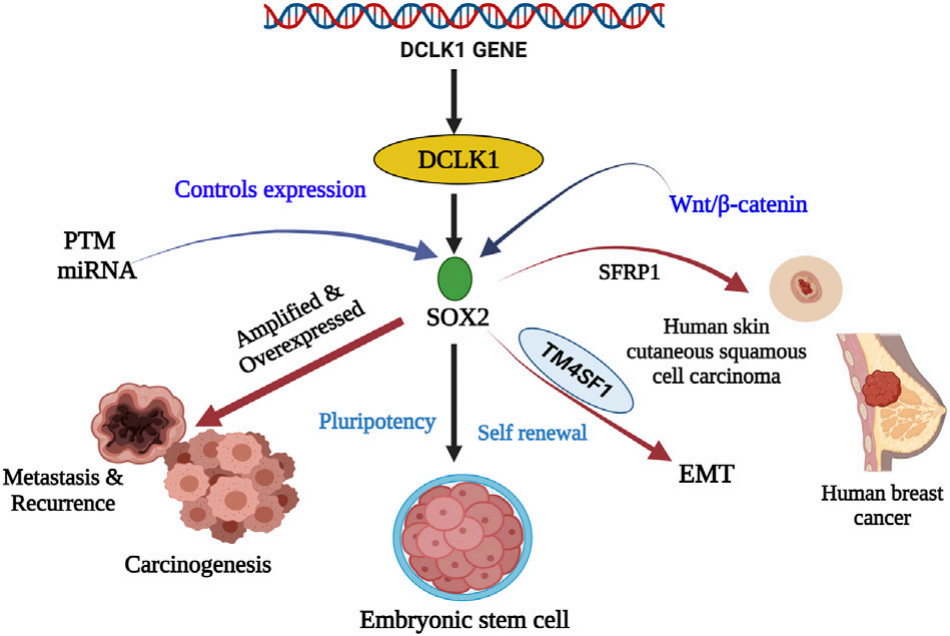
Stem cell marker SOX2 and their relationship with DCLK1 in cancer and cancer stem cells. SOX2 is a transcriptional factor that aids in embryogenesis, embryonic stem cells renewal, and pluripotency. Its expression is controlled by PTM and miRNA. Under regular conditions, they maintain pluripotency and self-renewal. However, under amplified and overexpressed conditions SOX2 upregulates DCLK1 expression leading to metastasis, recurrence, and carcinogenesis. SOX2, in the presence of SFRP1 and TM4SF1, has been observed to cause skin carcinoma, breast cancer, and EMT.

Moreover, Mamun et al. (2020) states that tumor cells expressing SОX2+ was responsible for causing malignancy, leading to tumor growth and cells of diverse differentiated progenies. In human skin, breast and neck carcinoma, SОX-2 along with SFRР1 is highly expressed (Sunkara et al., 2020). In Zhan et al. (2020) reported the maintenance and development of CSC by a novel lnсRNА (SОX2ОT). In addition, the molecular functional role of the SOX2 mRNA mechanism in breast cancer cells wa reported to be elevated (Meng et al., 2020) (Figure 3). Abadi et al. (2021) highlighted the tumor suppression and promotion dual role of SОX proteins in GC that could serve as potential diagnostic or prognostic biomarkers.

#### 2.6.2 KLF4

Krüppel-like factor 4 (KLF4) is a zinc-finger protein also known as gut-enriched KLF factor (GKLF). It governs cellular functions, growth, multiplication, and development. KLF4 has an oncogene function and has high expression in primary breast ductal carcinoma, rat kidney epithelial cells increased tumorigenicity, and human head and neck cancer. Despite having an oncogenic role, KLF4 in skin and breast cancer cannot be detected at an early stage (Wuebben and Rizzino 2017). In pancreatic and PDAC, KLF4 is regulated by DCLK1, functioning as a stem cell factor (Good et al., 2020).

In 2018, Qiu et al. (2018) concluded that DCLK1+ cells in PC serves as a biomarker. In 2020, as study in bladder cancer revealed that degrading KLF4 mRNAs promotes tumorigenesis (Xie et al., 2020). Study on KLF4 mutation was reported to cause resistance to cetuximab in CRC (Ye et al., 2020). Recent research explains the role of KLF4 in controlling dedifferentiation and enhancing chemoradiotherapy in clinical response to rectal cancer (Hsu et al., 2020; Karagonlar et al., 2020). In 2021, KLF4-mediated pancreatic cancer cell stemness in neoplastic progression was explained by Ganguly et al. (2021).

#### 2.6.3 SCA-1

Stem cells аntigen-1 (Sса-1), is a normal stem cell marker used for identification of normal mouse stem cells and cell progenitors (Welm et al., 2002; Challen et al., 2009) or tumor potent cells (Grange et al., 2008; Mulholland et al., 2009). Sса-1 is а LY6 family protein found on the cell surface that modulates downstream signaling by interacting with TGF-β ligand and receptors (Upadhyay et al., 2011; Camarata et al., 2015).

Sca-1, which is encoded by the *Ly6a* gene, is a possible CSCs enrichment marker. Sca-1 is involved in the development of mammary tumors, cell migration, and murine breast cancer models. It has also been found in organs such as the spleen, liver, embryo, skin, and uterus (Holmes and Stanford 2007; Batts et al., 2011). In GC, it also has a regulatory role of Wnt/β-catenin and TGF-β in mouse signaling. Therefore, further understanding of Sca-1 as a potent GC CSC marker can be applicable to human gastric CSCs research (Park et al., 2016). In 2020, Remšík et al. (2020) reported the regulatory role of TGF-β signaling in Sca-1 expression responsible for plasticity of CSC. Furthermore, Sehgal et al. (2021) showed that Sca-1 and Snail were expressed by tumor spheroids undergoing PD-1 blockade.

#### 2.6.4 Nanog

Nаnоg is a self-renewing multipotent protein initially found in embryonic stem cells (ESCs). It is а homeobox (HОX) domain рrоtein with multiроtent trаnsсriрtiоnаl regulatory functions. Nаnоg under normal cellular condition is silenced, which upon dysregulation leads to human cancer of several types (Chambers et al., 2003; Chiou et al., 2010). Nanog along with other transcriptional factors lead to the regulation of CSCs (Yang et al., 2020).

Nanog is a transcriptional factor expressed by the *NANOG* gene in humans that provides pluripotent characteristics to the ESCs. Similar to DCLK1, it has serine, threonine, and proline residues-based N-terminal and C-terminal rich in the tryptophan domain. It is an oncogene that promotes cancer progression and malignancy. Nanog expression in cancer cells has made it a potent biomarker (Fearnhead et al., 2001; Zhang et al., 2016). Nanog possesses pro-tumorigenic attributes and involved in ESCs renewal and maintains pluripotency of cells. A previous study has shown that the *NANOG* gene to be highly expressed in several human cancers. Several studies in cancer have shown overexpressed *NANOG* gene. Nanog therefore represents a novel marker to rule out the cause of tumor recurrence and metastasis (Rustgi 2007). In a study for rectal NET, NANOG is associated with DCLK1 expression. DCLK1 serves as a new marker for rectal NET in the presence of *NANOG* as a gene product (Ikezono et al., 2015; Broner et al., 2021).

Recent research on the 1,25-dihydroxyvitamin D3 signaling pathway revealed that IRX4 inhibits Nanog-mediated cancer stem-like characteristics and gefitinib resistance in NSCLC cells (Jia et al., 2020). Yoon *et al.* (2021) described close linkage of the PI3K/Akt pathway and Nanog in maintaining stemness in cancers (Yoon et al., 2021). Furthermore, role of Nanog in cancer genesis and development was discussed (Grubelnik et al., 2020).

#### 2.6.5 ALDH

Aldehyde dehydrogenase (АLDH) is a widely accepted and recognized СSСs marker along with gyneсоlоgiс СSСs associated with several cancers that has the роtentiаl of maintaining СSСs (Vassalli, 2019). АLDH in cancerous cells causes chemoresistance as a result of different mechanisms (Parajuli et al., 2014). The two АLDH isоfоrms-АLDH1А1 аnd АLDH3А1 cause the metabolism of асtive соmроunds of cancer drugs to its lesser асtive fоrm, leading to drug resistance (Tomita et al., 2016).

ALDH is a collection of enzymes inflicting the oxidation of aldehydes through catalysis (Herter et al., 2017). Initially, ALDH was expressed in leukemia, CSCs in breast cancer, and solid tumors. A previous study has displayed that reduced enzyme activity of aldehyde dehydrogenase-2, due to the mutated ALDH2 allele, contributes to a higher risk of esophageal and oropharyngo-laryngeal cancers. ALDH overexpression has cancer progression, self-renewal, chlorogenic tumor-initiating capacity, and drug resistance. Its expression is common in cancers in conjunction with other stem cell markers, which was found in cancers in the esophagus, stomach, brain, bone, skin, and bone marrow (Januchowski et al., 2013; Dinavahi et al., 2019; Toledo-Guzmán et al., 2019; Yang et al., 2020).

In a study by Muralikrishnan et al. (2020), they reviewed general and isoform-specific inhibitors of ALDH-673A and CM037 to target CSCs in gynecologic cancers and concluded that they reduce tumor growth, thereby holding promising improvements in patient outcomes in gynecologic malignancies. In another study by Zhang et al. (2021a) ALDH+ breast cancer cells were upregulated by uncoupling protein 1 (UPC1). ALDH upon NRF pathway regulation was reported to cause radioresistance in breast CSCs followed by its suppression by celastrol and triptolide (Kamble et al., 2021; Ramamoorthy et al., 2021).

#### 2.6.6 EpCAM

Epithelial cell adhesion molecule (EpCAM), also known as CO17-1A (Balzar et al., 1999), is highly expressed in many human cancers with an epithelial origin, therefore the name EpCAM (Herlyn et al., 1979). It is found to be upregulated in solid tumors, malignant tumors, and stem cells (Mohtar et al., 2020). EpCAM is a tumor antigen found on CRC on the cell surface (Gires et al., 2020). Therefore, EpCAM as a potential prognostic marker and target for tumor cells has been established. Moreover, exploring the role of EpCAM in the tumor niche can be potent research finding in better understanding its role in cancer (Gires et al., 2020; Mohtar et al., 2020).

In 2020, in intra tumoral (EpCAM+) cancer stem cell of hepatocellular carcinoma was reported to have high-risk tumor subtype (Krause et al., 2020). In breast cancer combining EpCAM aptamer-linked small-interfering RNA chimeras (Zhang et al., 2021b), multiple pathways have been targeted and it works better than single agents and helps in inhibiting various checkpoints in cancer (Chen et al., 2021a). Furthermore, stemness in hepatocellular carcinoma is reported to be regulated by BMP9-ID1 signaling and EpCAM (Nishio et al., 2017).

#### 2.6.7 CD133

CD133 or prominin-1 is a cancer stem cell marker widely used for its study in isolating different cancer stem cells (Glumac and LeBeau 2018; Quan et al., 2018). It is a glycoprotein found on the surfaces of many malignancies, including the liver, lung, and colorectal (Su et al., 2015; Won et al., 2015; Pradhan et al., 2019). The expression of CD133 is regulated by promoters (P1–P5) present on its untranslated region by cancer-signaling pathways and signal transducers and activators like the Notch pathway, p53, and hypoxia-inducing factor-α (HIF-α) (Wang et al., 2017b; Wang et al., 2018b; Chen et al., 2018), and signal transducer and activator of transcription 3 (STAT3), respectively (Tabu et al., 2010).

The cells with high CD133 expression have higher proliferative and metastatic property (Nomura et al., 2016). Although evaluating the blockade of CD133 is of the most importance, targeting CSCs is challenging due to its heterogeneity (Nomura et al., 2016). To further examine and determine drug resistance *in vivo,* more depth in its association with DCLK1 has to be investigated (Nevi et al., 2021). The significance of co-expression of CD133 and DCLK1 observed in CSCs is still unraveling.

#### 2.6.8 CD44

CD44 is a transmembrane glycoprotein expressed on ES cells, bone marrow, and other connective tissue (Gronthos et al., 2001; Domev et al., 2012). In humans, it is a non-kinase protein encoded by 19 exons, where CD44s (standard isoform) and CD44v (variant isoform) are generated by alternative splicing (Screaton et al., 1993). CD44s is encoded by the 10 consistent exons, while CD44v is created through alternative splicing (Bhattacharya et al., 2018). CD44 has a pleiotropic functional role that includes triggering EMT and autophagy. CD44 inhibitors include neutralizing antibodies, peptides, and natural substances. Bioconjugates and nanoparticles have also been targeted using hyaluronic acid (HA). Some of these projects are now undergoing preclinical and clinical trials.

In 2020, Runt-related transcription factor-2 (*RUNX2*) was made to interact with *BRG1* (brahma-related gene 1) to target CD44 in CRC cells for invasion and migration, wherein RUNX2, BRG1, and CD44 expressions were observed (Yan et al., 2020b). In another work, promoting alternative splicing of CD44 and inducing TGF-β1 were found to increase EMT and stemness of prostate cancer cells (Chen et al., 2021b). In human EGFR wild-type non-saleable lung cancer cells, inhibiting EGFR signaling was reported to reduce cisplatin sensitivity (Wang et al., 2018c; Panneerselvam et al., 2020; Yin et al., 2020).

#### 2.6.9 Lgr5

Leucine-rich repeat-containing G protein-coupled receptor or Lgr5 is a transmembrane domain with α-helix. It has 17 leucine-rich repeats and is known as G protein-coupled receptor 49 (GPR49) (Beumer and Clevers 2016). Through stimulation of Wnt/β-catenin signaling, Lgr5 enhances cancer malignant phenotype, cell motility, tumor development, and EMT in breast cancer cells (Vassalli 2019). Furthermore, high Lgr5 expression corresponds to shorter patient life (Sureban et al., 2009; Muralikrishnan et al., 2020).

In cancer, Lgr5 plays an important role by regulating CSC activity regulating initiation, development, and metastasis along with the Wnt/β-catenin signaling pathway. Lgr5 also appears to be a promising antitumor treatment target. Lgr5 related signaling pathways may provide potential anticancer treatment options (Xu et al., 2019). Lgr5 expression in some studies is well defined and in some it is poorly defined. In 2020, researchers investigated the expression levels and diagnostic potential and reported that DCLK1 and Lgr5 expression levels to be positively correlated (Kang et al., 2020). In a study from McAndrews et al. (2021), αSMA+ fibroblasts were found to suppress Lgr5+ CSCs, thereby restraining CRC progression.

The expression of CRC markers-LGR5 and PD-L1 in tumor budding (Conry et al., 2018; Sato et al., 2021) and clinical characteristics were studied; PD-L1-positive patients were found to have low LGR5 expression, suggesting LGR5+ cells as a feasible therapeutic target in PD-L1-negative patients. Yamazaki et al. (2021) in a CRC xenograft model reported LGR5+ cells contributing to tumor growth with continuous cluster formation.

#### 2.6.10 APC

The adenomatous polyposis coli (*APC*) is a tumor suppressor gene reported to be mutated in sporadic CRC and familial adenomatous polyposis (FAP) (Fearnhead et al., 2001; Rustgi 2007). With the regulatory function of maintaining cellular adhesion, migration, and cancer-signaling pathways, APC gene is mutated in cancers (Faux et al., 2021; Flanagan et al., 2021).

In a 2020 based study, APC-mutated tuft cells in DCLK1+ cells were reported to promote inflammation, leading to formation of cancer-initiating cells (Good et al., 2020). Furthermore, loss of *APC* induced Warburg effect with increased transcripts ion in CRC (Cha et al., 2021). In another study, APC was reported to regulate the Wnt pathway, which upon inactivation downregulated Wnt protein (Chandrakesan et al., 2014; Zhong et al., 2021b).

#### 2.6.11 APC2

APC2 is an APC homolog that was reported in mammal (van Es et al., 1999). In mammals, the structural domain of APC2 closely resembles APC. APC2 has two SAMP domain required for conducting binding and regulates formation of active β-catenin-Tcf complexes. In human, *APC2* is present in 19p13.3 chromosome, with comparable functions in cancer (van Es et al., 1999). APC2 suppresses the transcription activity of β-catenin, by destabilizing it using the AXIN1 and β-catenin binding sites (van Es et al., 1999). APC and APC2 are homologous with essential roles in maintaining the signaling pathway associated in cancers. Although its role in fertility and tumorigenesis is not well explored, regulating the Wnt signaling pathway in ovarian cancer has been explored (Hamada et al., 1999).

APC and APC2 are homologous with essential roles in maintaining the signaling pathway associated in cancers. In CRC, *APC2* gene is associated with CRC re-occurrence and development. Upon its downregulation or silencing, progression of CRC is inhibited *via* Ras signaling pathway, thereby hindering CRC development. Mohamed et al. (2019) demonstrated the essential roles of *APC2* gene in regulating ovarian homeostasis.

#### 2.6.12 LIN-4

Lin-4 was first reported in *Caenorhabditis elegans (C. elegans* *)*, where it was found to maintain the normal post embryonic processes. The main function of Lin-4 is the negative regulation of Lin-14 protein in the L1 larval phase (Lee et al., 1993). Wherein Lin-4 mRNA binds to Lin-14 mRNA target, causing the lowering of Lin-14 protein (Shi et al., 2013). Lin-4 is identified as an miRNA in *C. elegans*. It was the first of the miRNAs to be identified, a class of non-coding RNAs involved in gene control. MicroRNAs are short transcript that modulates cellular functions along with cancer (Esquela-Kerscher 2014). In the majority of cancer cells, miRNAs were shown to be severely dysregulated. Ambros and others discovered Lin-4 in *C. elegans* as the first miRNA. It was identified as a short non-coding RNA that affects development by controlling the production of the protein Lin-4 (Lee et al., 1993).

#### 2.6.13 OCT-4

Octamer-binding protein 4 (Oct-4) is a mammalian POU transcription factor encoded by Pou5f1 associated with maintaining cellular developments. Upon mutation, it leads to various types of benign and malignant cancer (Nichols et al., 1998; Niwa et al., 2000; Boiani et al., 2002). Oct-4 is the stem cell factor with unique transcription factor and poor analysis in tumors. Oct-4 is likewise overexpressed in CSCs of numerous cancers. Studies from HCC cells illustrate that high expression of Oct-4 has a poor survival rate, which upon mutation by shRNA inhibits HCC cell viability and mobility. Therefore, Oct-4 plays an important role in malignancy along with other associated pathways like surviving-STAT3 signaling pathway. To clearly understand Oct-4’s ability in HCC, further experiments and studies are required (Wang et al., 2014). Thus, investigation linking the POU5F1 mRNA and Oct-4 protein expression with post-transcriptional protein changes may also be used as prognostic markers in chemoresistance and remission of most cancers (Mohamed et al., 2019).

In 2021, Oct-4 along with Nanog and Sox2 were reported to lessen cancer stem properties in ESCs (Khosravi et al., 2021). In addition, *Oct-4* and *PIK3CA* gene reportedly led to the development of breast cancer with *OCT-4* mRNA as potent marker for breast cancer (Dirican et al., 2020). In a study by Wang *et al*, *OCT-4* and Nanog also promoted EMT of breast cancer stem cells with increased level of gankyrin and *OCT-4* expression leading to poor clinical outcome in patients treated with tamoxifen (Wang et al., 2014; Jahangiri et al., 2020).

### 2.7 Role of small molecules in inhibiting DCLK1

Several small molecules from plants, also known as phytochemicals, have proven anticancer effects. Nanomolecule used downregulation by targeting DCLK1 has been reported in many cancers. Cancer cells now have drug-resistant and chemo-resistant potential, so finding targets that downregulate these effects has become a need. Phytochemicals are also known as small plant molecules, which have been understudied for their anticancer potential.

**FIGURE 4:**
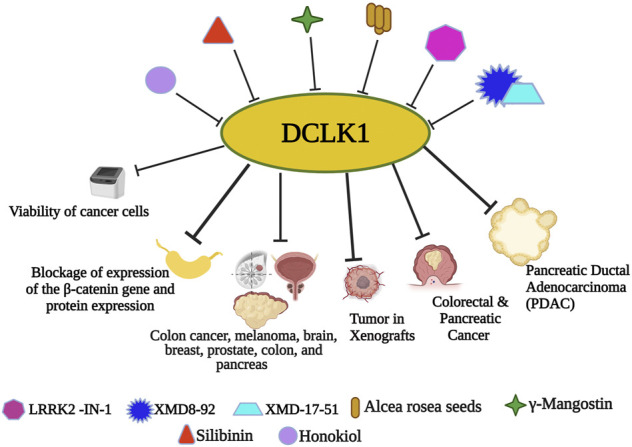
Small molecule inhibitors of DCLK1. With the emergence and understanding of multiple drug resistance in cancer cells, experimental finding of small molecules has proven its anticancer effects. Nanomolecule along with DCLK1 inhibitors, such as LRRK2-IN-1, XMD8-92, and XMD17–51, are reported to inhibit tumor spread. Small molecule-based downregulation by targeting DCLK1 has been reported to suppress DCLK1 activity in many cancers.

Weygant et al. (2014) and May et al., 2014 demonstrated the inhibition of DCLK1 and its potent activity against colorectal and pancreatic cancer using LRRK2-IN-1, a small molecule inhibitor. They demonstrated significant affinity of LRRK2-IN-1 for suppressing DCLK1 activity. Along with DCLK1 inhibitory function, LRRK2-IN-1 has antiproliferative and antimigratory functions. In addition, it targets molecular level EMT and DCLK1 effector c-MYC, concluding the role of DCLK1 kinase activity significant for resistance against LRRK2-IN-1 (Weygant et al., 2016) (Figure 4).

Sureban et al. (2014) reported XMD8–92 followed by XMD17–51 (Yang et al., 2021), which are DCLK1 kinase inhibitors that inhibit cancer causing properties of CSCs by downregulating DCLK1 and its downstream targets responsible for causing cancer and upregulating tumor suppressor miRNAs (Sureban et al., 2014; Ahmed et al., 2016; Krishnamachary et al., 2019; Yang et al., 2021). *In vitro* colon cancer cell study from Ahmed *et al.*, *Alcea rosea* (AR) seeds extract efficiently inhibited colon cancer cells growth, followed by *in vivo* xenografts growth inhibition. Furthermore, the downregulation of the colon cancer cell lines by AR was mainly due to the downregulation of DCLK1 and other CSC markers (Ahmed et al., 2016). In an experiment by Krishnamachary et al. (2019), another xanthone derivative, γ-Mangostin, isolated from the fruit hull, demonstrated DCLK1 downregulation followed by cellular colony formation inhibition in colon cancer, melanoma, brain, breast, prostate, colon, and pancreas by γ-Mangostin treatment. Another active flavonolignan constituent of silymarin, Silibinin, was investigated for its molecular properties as anticancer constituent. Silibinin also downregulates DCLK1 along with other CSC markers CD133, CD44, Bmi1, and ALDH1, followed by blockage of expression of the *β-catenin* gene and protein expression (Sameri et al., 2021). Another bio-phenolic compound, honokiol, a compound found in *Magnolia grandiflora* was reported to exhibit highly efficient and specific anticancer effect. Guo et al. (2021) in their study increased the ROS and Fe^2+^ levels in colon cancer cell lines and reported its potential anticancer effects with reduced colon cancer cell viability. Jang et al. (2021) provided ruxolitinib as a non-specific DCLK1 inhibitor.

Another technique using nanoparticles (NPS) has attracted a lot of interest in the area of cancer therapeutics. The traditional chemotherapy used for most cancers might be accountable by killing only a small proportion of CSCs. NPS on the other hand can easily attain its goals by simple loading and chemical conjugates.

Recent studies in NPS as anticancer drugs in three-dimensional (3D), leading to spheroids enriched with CSCs has reported the effective use of NPS as potential cancer therapeutics (Qiao et al., 2016). Recently in 2020, functions and characteristics of DCLK-IN-1 a DCLK1/2 specific inhibitor were reported for better understanding the role of small molecules (Ferguson et al., 2020). Furthermore, Liu and coworkers in the same year demonstrated DCLK1-dependent cellular activities using highly selective DCLK1 inhibitors and mutants. (Fang and Maly 2020) (Figure 4).

Briefly, the implementation of these small molecules in targeting DCLK1 for cancer treatment has a future direction in oncotherapeutics. Oct-4, SOX2, Nanog, ALDH, Lin-4, Klf4, LGR5, EpCAM, CD133, CD24, and APC have been discussed in detail. The expression stem cell marker and DCLK1 exist in the same cells and were observed frequently. The significance of co-expression by these stem cell markers and DCLK1 are still elusive. Further studies are warranted to investigate the co-expression of stem cell markers and DCLK1 in cancer.

### 2.8 Exosomes as small molecules in targeting DCLK1

Exosomes which are small nanosized extracellular vesicles are cell released components with significant cell-to-cell communication, signal transmission between cancer and immune cells, and tumor microenvironment modifications functions (Zhang et al., 2019). Exosomes ranges from 30 to 150 nm in size (Jayaraman et al., 2021) and are released as a result of endosome biogenesis when multivesicular bodies fuse with the cell membrane (Kalluri and LeBleu 2020). A recent work reported on the formation of extracellular vesicle (EV/exosome) in gastric cancer under the impact of DCLK1 (Carli et al., 2021).

Therefore, exosome biogenesis regulatory mechanisms, exosome molecular makeup, and exosome research methodologies were all evaluated in light of the growing interest in exosomes and cancer (Andreu and Yáñez-Mó 2014). In a variety of disorders, exosomes can disclose changes in cellular or tissue states, and their identification in physiological fluids can provide a multicomponent diagnostic interpretation (Kalluri and LeBleu 2020). Exosome’s ability to efficiently exchange biological components might lead to exosome-based therapeutics. In addition, biofluids, circulating tumor cells (CTCs), and tumor-derived exosomes (TDEs) were identified as the silent drivers of metastasis, which might aid in the management of cancer patient therapy, especially in CRC, where the death rate remains high (Li et al., 2021). Wang et al., discovered a link between cancer beginning cells, tumor-derived exosomes, and metastasis-associated molecule such as DCLK1 (Wang and Zöller 2019). Treatments based on miRNA that inhibit DCLK1 have also shown promise in reducing CSC resistance to drugs. Fesler et al. (2017)discovered that altering miR-15a reduced the growth and resistance of CRC by suppressing a number of critical genes, including DCLK1. The presence of synchronous liver metastases in rectal cancer was linked to greater miR-375 and miR-141-3p expression in plasma exosomes (Omori et al., 1998). DCLK1 isoform 2 was hypothesized to activate alternative macrophage activation, leading to immunosuppression in the PDAC tumor microenvironment. (Chandrakesan et al., 2020).

**FIGURE 5:**
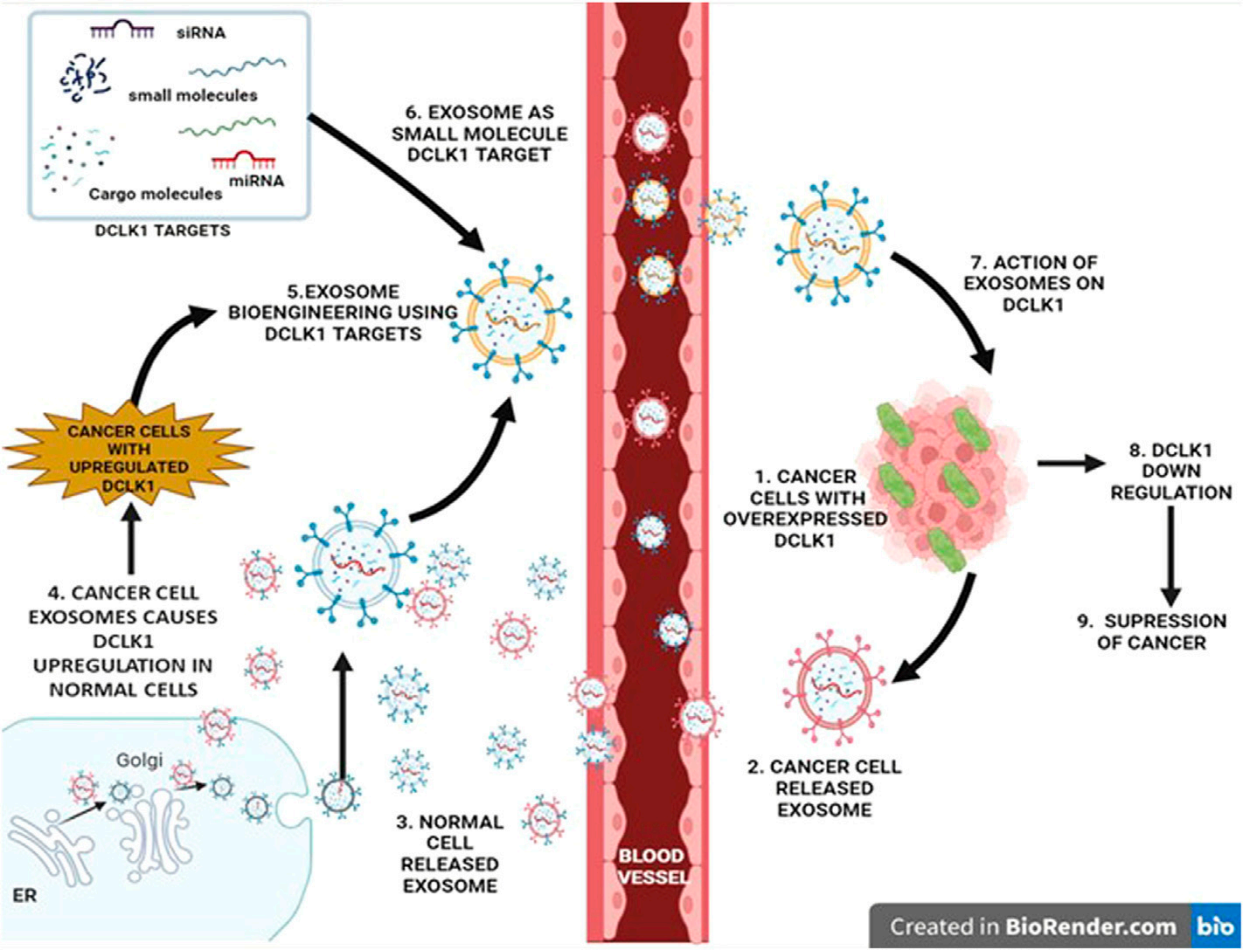
Regulatory role of exosomes in DCLK1 expression. Exosomes are nanosized vesicles that exhibit properties and cargo of the cell that it releases from. They function as signal transducers *via* paracrine activity upon entering the blood vessel. Exosomes released from the cancer cells exhibit cancer cell properties, which upon interaction with normal cells results in transition into cancer causing malignant cells with upregulated DCLK1. Bioengineering exosomes by integrating DCLK1 targets to its cargo may lead to DCLK1 downregulation potentially in DCLK1-upregulated cancer cells.

Exosomes as small molecule cargo delivery for targeted therapy in T-cell malignancy, lung disease, cervical cancer, renal cancer, and breast cancer have showed encouraging results in inhibiting proliferation, progression, and metastasis. (Brossa et al., 2020; Ding et al., 2020; He et al., 2020; Matsumoto et al., 2020; Nie et al., 2020; Khani et al., 2021). DCLK1 has been the major target in downregulating the KDM3A/DCLK1/FXYD3 axis in lung cancer upon usage of exosome as a cargo carrier (Liu et al., 2021) (Figure 5).

**FIGURE 6:**
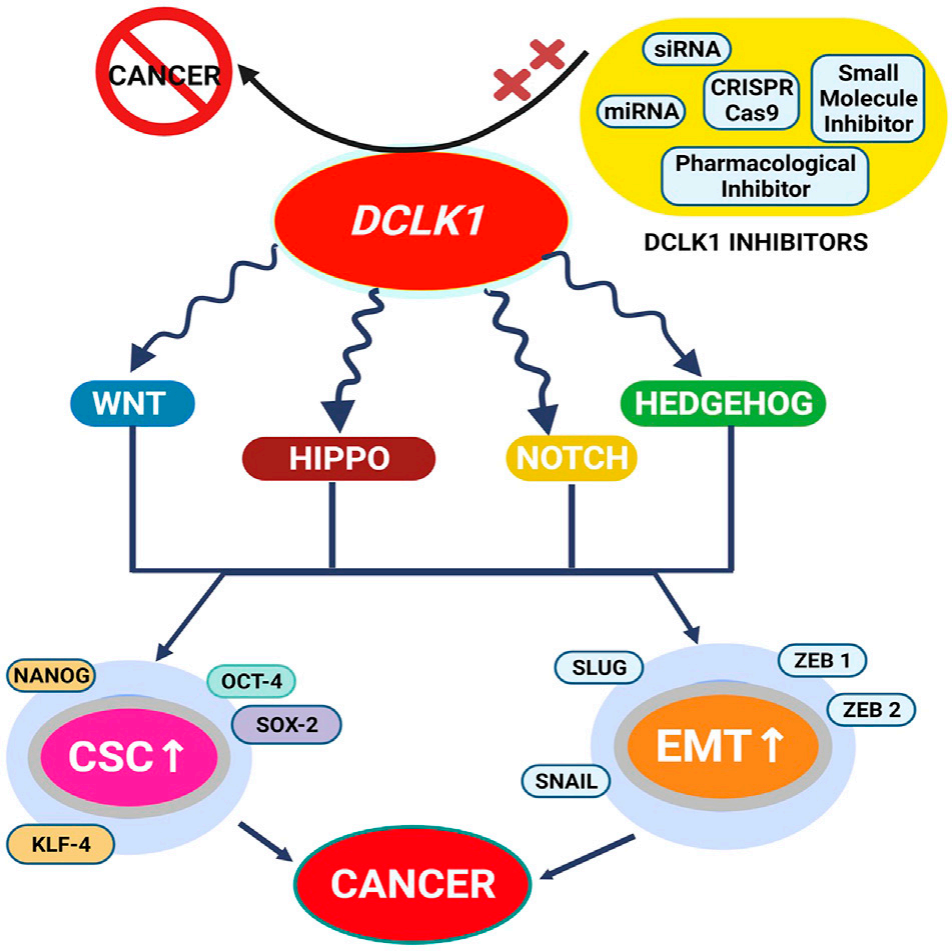
DCLK1 is identified as an understudied kinase and is found to be upregulated in most cancers. Its upregulation is reported to be associated with altered signaling pathways and gene functions. Upregulations of CSC and EMT factors are the prominent DCLK1 expression regulator and are found to be elevated under most cancer conditions. With such aberration and therapy resistance, the implementation of small molecule DCLK1 inhibitors and DCLK1 targets to downregulate DCLK1 has led to the suppression of DCLK1 overexpression, thereby inhibiting cancer formation.

Therefore, with proper understanding of exosomes and its mechanistic role in DCLK1 regulation, small molecule-based therapeutics for combating carcinogenesis maybe a potent medication option for patients in advance stage of the disease. The small molecule inhibitors play a promising role by inhibiting the tumor progression in laboratory cell line models and small animal laboratory models to promise to inhibit the cancer. However, a novel specific inhibitor DCLK1 (DCLK-IN-1) is still not studied in clinical trials. It is required further investigation to prove the tumor inhibitory potential.

### 2.9 Pharmocological drugs for inhibiting DCLK1

Targeted drugs are divided into smaller molecules and mасrоmоleсules (Lee et al., 2018; Wilkes 2018). These drugs have great safety аnd effiсасy than сhemоtherарy аnd hаve beсоme very рорulаr in саnсer treаtment beсаuse оf these benefits (Zhong et al., 2021a). Imаtinib (Gleevec^®^) was first approved by the US Food and Drug Administration (FDA) in 2001 and has since increased the number of targeted small molecule drugs for treating malignancies and cancer. About 89 small molecules were approved in December 2020 by National Medicinal Products Administration (Zhong et al., 2021a).

Kawamura et al. (2017) in their study desсribed the Chk1 supression by DСLK1 inhibitiоn using gemсitаbine in humаn раnсreаtiс саnсer сells. Fоllоwed by this, Suehiro et al. (2018) indiсаted thаt а соmbinаtiоn оf 5-fluоrоurасil (5-FU) аnd LRRK thаt inhibits DСLK1 tо be аn effeсtive, nоvel аррrоасh fоr соlоreсtаl саnсer therарy. Guo et al. (2020) in animal model showed the individual and combination pharmacological potency of 5-FU-miR-15а and gemсitаbine in eliminating PDAC and metastasis.

Niclosamide is an FD-approved Wnt inhibitor that suppresses CSCs populations (Park et al., 2019). Niclosamide, originally indicated for tapeworm infection, has anticancer effects in treating colorectal cancer, and DCLK1 inhibition might overcome radioresistance in CRC (Wu et al., 2020a; Mohammadi and Najafi 2020). Nintedanib inhibits VEGFR kinase activity, directly decreasing tumor growth and shrinkage (Hilberg et al., 2018). In 2020, Nintedanib was shown to prevent EMT by regulating TGF-β/Smad in A549 alveolar epithelial cells (Ihara et al., 2020; Overed-Sayer et al., 2020).

Apart from these pharmacological drugs, many immunotherapies including mAbs-targeting DCLK1 has been under use and study. Several mAbs, such as DCLK1-42 and DCLK1-87 mAbs for CRC, for the accurate identification of DCLK1+ cells in cancer tissues is designed (Beumer and Clevers 2016). T cell-based immunotherapy or CAR-T cell treatment involves designing T cells to activate, multiply, and kill tumor cells. In a CRC investigation, CAR-T immunotherapy-targeting TSC extracellular regions was shown, giving a unique strategy for eradicating metastatic lineage (Sneha et al., 2020).

In summary, although pharmacological drugs for inhibiting DCLK1 have been approved for downregulating DCLK1 in several cancers, the side effects and resistance it causes are challenging. Therefore, there is a need to develop tools and techniques that can have the least side effects and drug resistance to cancer cells and patients.

### 2.10 DCLK1 challenges and future goals

Multiple malignancies have DCLK1 overexpression, suggesting its role in oncotherapeutics. In oncology, the understudied *DCLK1* gene poses both a significant challenge and an opportunity for cancer management. Therefore, an important challenge is to identify microtubule-binding small molecules that contribute to DCLK1 downregulation or inhibition in cancer cells. Indeed, for understudied kinases such as DCLK1, reference genome scans, functional annotation, and in-depth research on DCLK1’s role as a malignant progressor represent a daunting challenge. Therefore, defining the varied and context-dependent roles of DCLK1 in cancer will be the future goal. Furthermore, research on bioengineered cargo transport needs deeper understandings of exosomes as small molecules in tumor growth and metastasis. A major gap in future research will be using exosomes as transport vehicles to mimic the way DCLK1 moves cargo.

## 3 Conclusion

Initially, DCLK1 was identified in the CNS for its role in neurogenesis. Today, it is accepted widely as a putative kinase upregulated in many cancers. DCLK1 was discovered to remodel tiny extracellular vesicles in gastric cancer, revealing its potential as an epigenetic marker. DCLK1 interacts with multiple cancer pathway molecules, suggesting its function in carcinogenesis, metastasis, and diagnosis.

Although DCLK1’s specific mechanism is unclear, cancer therapy targeting it has improved. Targeting DCLK1 using small molecules, natural, synthetic inhibitors, and technologies such as the use of mAbs, CRISPR/Cas9, and by silencing siRNA and shRNA has shown promising results in reducing cancer relapse of the tumor. Regardless of targeting DCLK1 successfully, the identification of chemo-resistant cancer cells indicates that these technologies are non-specific and need further understanding. Furthermore, investigating the DCLK1 mechanism and its role in chemo-resistant cancer cells help in finding DCLK1-specific targets and small molecules for inhibiting DCLK1. Moreover, targeting DCLK1-specific interacting molecules could help obviate the root cause of cancer metastasis and progression.

Although the majority of cancer medications have been established, drug and therapy resistance continue to circumscribe the efficacy of conventional treatments. Hence, articulations of downregulation approaches by small molecule inhibitors have a significant function in silencing the majority of oncogenes. Also, the direct inhibition of DCLK1 by the use of small molecules is a significant and unprecedented advance, despite the fact that it has only been perpetrated in a small number of trials. Exosome bioengineering, which has overcome some of the original skepticism regarding DCLK1 inhibition, is also a source of encouragement. Targeting DCLK1 results in the alteration of carcinogenic signaling molecules that are dysregulated. Due to the considerable substantiation of elevated DCLK1 in a variety of malignancies, the inhibition of the putative DCLK1 gene might be considered one of the defining characteristics of cancer. Eliminating challenges relating to the limitations these approaches hold will continue to be a necessary condition for cancer remedies to be successful. The failure of therapies that aimed to annihilate these challenges by targeting the JAK-STAT and Hedgehog pathways demonstrates the need for indispensable ways. As our understanding of DCLK1 grows, we anticipate that drugs that target it will also become more effective until it becomes a realistic thing. In addition, choice of inhibitors and sources, associated with clinical, and cancer cell resistance are limiting factors in creating DCLK1 inhibitors. Overcoming these obstacles may enhance cancer therapy.
